# Psychometric properties and factorial structure of the Spanish version of the psychological capital scale in Ecuadorian university students

**DOI:** 10.1371/journal.pone.0285842

**Published:** 2023-05-25

**Authors:** Víctor López-Guerra, Karina Ocampo-Vásquez, Lucía Quinde, Sandra Guevara-Mora, Jesus Guerrero-Alcedo

**Affiliations:** 1 Department of Psychology, Universidad Técnica Particular de Loja, Loja, Ecuador; 2 Faculty of Health Sciences, Universidad Científica del Sur, Villa EL Salvador, Perú; University of Valencia: Universitat de Valencia, SPAIN

## Abstract

**Background:**

Psychological capital (PsyCap) as a higher-order positive psychological resources (that include hope, efficacy, resilience, and optimism, or the HERO within). This construct was widely described and evaluated in the workplace; however, there is little research in other contexts, such as education, due to the lack of validated and adapted instruments in Latin America. Therefore, the objective of this study is to analyze the psychometric properties and factorial structure of the Spanish version of the psychological capital scale in a large sample of Ecuadorian university students.

**Methods:**

A non-probabilistic convenience sample of 1732 university students (mean age 20 years, SD = 2,29; 55% female) from the city of Loja—Ecuador were surveyed online using a cross-sectional design.

**Results:**

The respecified second-order 4-factor model showed the best fit to the data (CMIN/DF = 7.99, CFI = .977, TLI = .970 NFI = .974, IFI = .980, AIC = 443.833, RMSEA = .064 [058, .070]), and such model remained invariant across sex, age and public and private institutions. The internal consistency was adequate, with Alpha and Omega coefficients for the total scale (α = .941, ω = .942) and its four factors: self-efficacy (α = .869, ω = .872), hope (α = .888, ω = .889), resilience (α = .774, ω = .785), and optimism (α = .840, ω = .840). Finally, the PsyCap and its dimensions correlated with academic engagement and satisfaction.

**Conclusions:**

The psychological capital showed adequate psychometric properties in university students, and its use in this context is supported.

## Introduction

Psychological capital (PsyCap) has its origin in positive psychology from a focus on the personal strengths and psychological capacities of the human being that can be developed and enhanced, instead of emphasizing only trying to "fix" mental illness. PsyCap refers to a state of positive psychological development or personal development that is made up of four dimensions: self-efficacy, optimism, hope and resilience [1]. Self-efficacy consists of having self-confidence and mobilizing one’s cognitive resources, motivations, actions and abilities to achieve success in challenging tasks [2, 3]. Optimism lies in making positive attributions about present and future events, considering negative situations as temporary and avoiding generalizations [3, 4]. Hope is a state of positive motivation, made up of perseverance until the fulfillment of objectives and planning or reorienting of one’s trajectory if necessary to succeed [5]. Finally, resilience consists of maintaining or recovering from problematic or adverse situations to achieve success, and it is characterized by accepting reality, believing that life is significant as well as adapting to changes [3, 6, 7].

The instruments to measure PsyCap were created to evaluate it within the workplace, initially using indirect methods through speech or writing samples; however, due to the long time spent processing information, this measure was not relevant within the investigations of PsyCap. After that, Luthans and collaborators created in 2007 the psychological capital scale. Their psychometric study was carried out with four independent samples, and the authors found that the factorial structure of the psychological capital scale with the best fit to the data is the second-order model with four factors (hope, efficacy, resilience and optimism). In general, they achieved optimal levels of internal consistency in the total scale (.88, .89, .89, .89) and in each of its dimensions, except for the optimism factor in the second sample (.69) and for resilience in the third sample (.66). Likewise, in the second study, they found that psychological capital as a higher order factor is positively related to both performance (r = .33, p < .01 in the manufacturing firm and r = .22, p < .01 in the service firm) and satisfaction (r = .32, p < .01 in the manufacturing firm and r = .53, p < .01 in the service firm) [8]. Thus, the scale proposed by Luthans has become the most widely used worldwide to measure this construct in the workplace, and it is available in several languages [1, 9], including Spanish [10, 11], Portuguese [12], Turkish [13], and Italian [14].

Although it is true that research on psychological capital and the scale to assess it has focused mainly on the occupational context [10, 11, 14–17], there is evidence that it can also be applied in the academic field [18, 19]. It should be noted that, within this context, research has focused on the study of each dimension separately, but not on their combination as a construct [19]. Therefore, there is little literature in this area [20–22]. In this sense, there is evidence that individuals with high psychological capital (considering its four dimensions) tend to present greater satisfaction and performance than if the dimensions are considered separately, and there is empirical evidence that the combination of these four dimensions generates synergy, making it in a high-level construct [1, 8].

In view of the growth in the use of this scale, the need to adapt it to the academic context and the Spanish language started to become visible, in order to measure its association with positive results in students. Thus, there are few studies regarding this context. The first adaptation of the scale to the academic context and translated into the Spanish language was proposed in 2019 by Martínez and collaborators. Their adaptation consisted of 12 items and was validated in two samples, one of Spanish students and the other with Chilean students, where they found an α = .80 and .89, respectively, and similarly an ω = .80 and .89. Likewise, in terms of construct validity, adequate fit indices were found for a second-order model with four factors. In terms of criterion validity, the results in both samples indicated a positive correlation between PsyCap and academic engagement and satisfaction, with r values ranging from .39 to .45, and academic performance, with r values ranging from .16 to .18 with a p < .001. In this way, it was shown that this version in Spanish and adapted to the academic context is a shorter and more reliable tool to be used by researchers and professionals in the area [20]. Likewise, adaptations have been developed in the educational field in other countries such as Argentina, where the scale obtained a ω = .93 and factor analysis shows a first-order model with four factors. For convergent validity, PsyCap was related to academic engagement and the student’s grade average, with the results showing that there is a positive correlation between the 4 dimensions of PsyCap (self-efficacy, hope, resilience and optimism) and the three dimensions of engagement or academic commitment: vigor, dedication and absorption [18].

Based on the aforementioned, divergences are reflected in the factorial analysis of the PsyCap scale in Latin America. Then, considering that the properties of the scales can be affected by cultural factors and that in Ecuador there is still no evidence of a Spanish version allowing the evaluation of psychological capital and its possible applications in obtaining positive results at the academic level in students, the objective of this study is to analyze the psychometric properties and the factorial structure of the psychological capital scale adapted to Spanish in a different context, such as the academic, in Ecuadorian university students.

## Materials and methods

### Participants

A non-probabilistic and convenience sample of college students enrolled in three universities of the city of Loja–Ecuador: Private Technical University of Loja, National University of Loja and International University (Loja campus) were invited by email or mobile phone to participate in the study, and they then completed a computerized survey. A total of 1732 participants (792, 753, and 187 from each University, respectively) met the inclusion criteria of being enrolled in, at least, one whole academic year, and completing the entire survey (the average response rate across universities was 42%). The mean age was 20 years (SD = 2.29), and 953 (55%) were female, 779 (45%) male, 97% single, 57% from private universities and 94% without children. Participants received no economic compensation for their participation.

### Measures

#### Psychological Capital Questionnaire in Academic Context (PCQ-12)

Instrument that was adapted to the academic context and assesses psychological capital through four dimensions: hope, self-efficacy, resilience and optimism. It consists of 12 items (3 of self-efficacy, 4 of hope, 3 of resilience and 2 of optimism), in which the participant responds to a Likert-type scale with scores between (1 = totally disagree) and (6 = completely agree) that are interpreted on a continuum where higher scores mean that the person evaluated has a higher level of psychological capital. An example item is the following: "Currently I think I am having a lot of success in my studies." Regarding the psychometric properties, a study was carried out on two samples of students, and a second-order model with four factors was evidenced, showing adequate internal consistency for each of its factors (hope [α = .78 –.83], self-efficacy [α = .70 –.75], resilience [α = .58 –.68], optimism [α =. 62- .76]) and presenting an ω = .80 to .89. In terms of criterion validity, the results indicated a positive correlation of PC with the dimensions of engagement and academic satisfaction [20].

#### Academic satisfaction scale (ASS)

It is made up of 7 items that constitute a single factor that measures the well-being and enjoyment that students perceive in relation to their role as such. This instrument is presented in a 7-point Likert format that varies from (1 = totally disagree) to (7 = totally agree). An example item is: “I enjoy my classes most of the time.” Regarding the psychometric properties, it presents a satisfactory internal consistency with an α = .917 and a one-dimensional factorial structure [23].

#### Academic engagement

This instrument presents 9 statements that represent manifestations of vigor, absorption and dedication to studies. An example item is "My tasks as a student make me feel full of energy." It must be answered based on frequency of time from alternatives in a Likert format that goes from (0 = never) to (6 = every day). Its psychometric analysis indicates that this scale is made up of two factors such as predisposition to study and satisfaction with studies, and both have an Alpha coefficient of .87. When analyzing the correlations between both, a direct and significant relationship was evidenced (r = .69, p < .001) [24].

#### Design and procedure

A cross-sectional study was conducted. The data collection was carried out for 5 months. The participants were sent, by email or mobile phone, a link to google forms that allowed them access to the informed consent form and the objectives of the study.

This study was conducted in accordance with what was expressed in the Declaration of Helsinki by Mazzanti Di Ruggiero (2011), in its updates, and in the current codes such as: non-discrimination, consent, confidentiality, personal data protection, gratuity, and having the power to withdraw at any time and without any type of repercussions [25]. In addition, the students were informed that their participation was voluntary, confidential and anonymous. In case of accepting the informed consent, the students proceeded to answer the psychological scales.

#### Data analysis

The statistical analysis was carried out using the IBM Statistical Package for the Social Sciences (SPSS) software (IBM Inc., Chicago, IL, USA; version 26.0), AMOS version 25.0 (IBM Inc., Armonk, NY, USA).

Firstly, for the Confirmatory Factor Analysis (CFA), the maximum likelihood method was used, and the estimators of the goodness of fit were the minimum discrepancy per degrees of freedom (CMIN/DF), the Bentler comparative fit index (CFI), Tucker Lewis index (TLI), the Bentler–Bonett normed fit index (NFI), incremental fit index (IFI), Akaike information criterion (AIC), and the root mean square error of approximation (RMSEA) and its confidence intervals (CI: 90). Good fitness of the model was considered if CMIN/DF < 5; CFI, TLI, NFI, IFI > 0.900 (better if > 0.950).; and RMSEA < 0.080 (better if < 0.060) [26]. AIC was used to evaluate alternative models with the smaller value in each case indicating the best fitting model.

**Fig 1:**
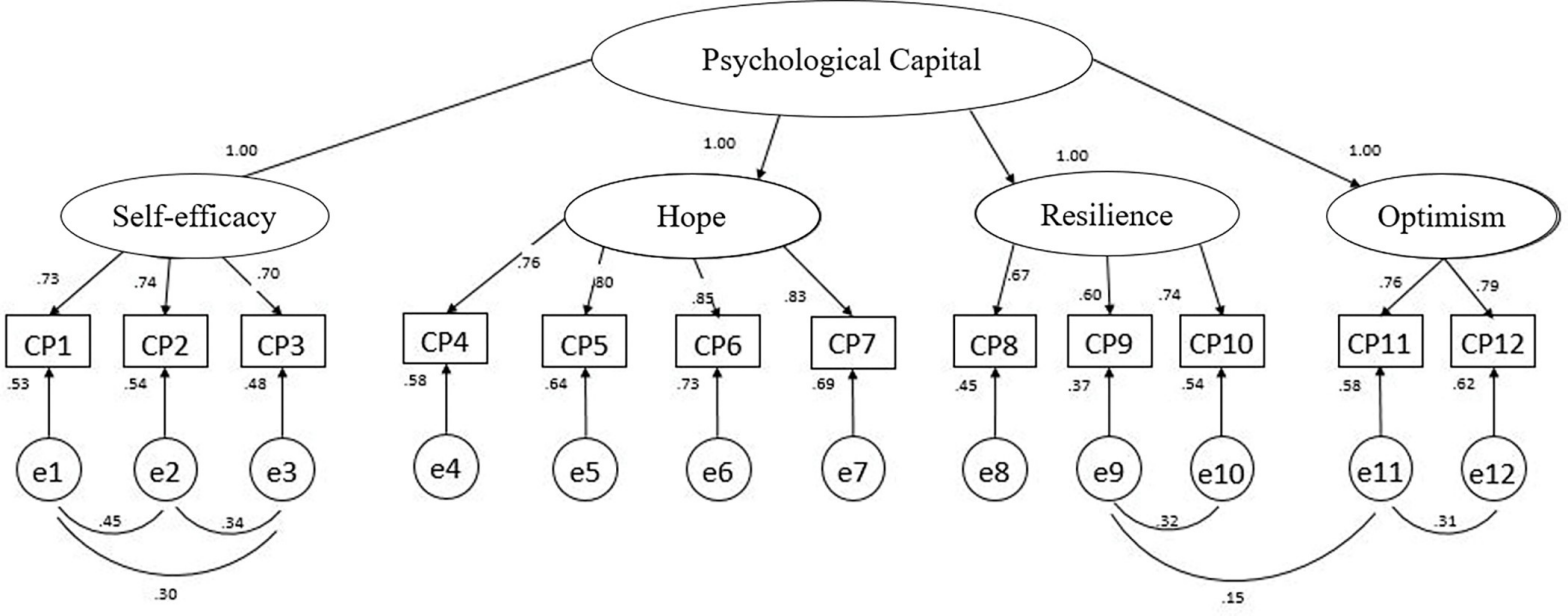
Respecified second-order 4-factor model.

According to precedent literature using a CFA approach, we compared in our study the fit of four distinct and possible models of the Ecuadorian version of PCQ-12 (see Fig 1). Those are Model 1: a unidimensional structure where each item is represented by a one-factor, therefore including the twelve items assessed within a unique general factor [27, 28]; Model 2: a first-order model where the items are grouped into four factors, namely self-efficacy (includes items 1 to 3), hope (includes items 4 to 7), resilience (includes items 8 to 10), and optimism (includes items: 11 to 12); Model 3: a second order model where the psychological capital construct is represented by 4 dimensions as proposed by the original author [29]; and Model 4: similar to model 3 but taking into account the covariations between the errors of the different items [20].

Secondly, we assessed the factorial invariance of the PCQ-12 across the total sample, taking into account the following models: configural invariance (Model configural, MC), which indicates a factorial structure without restrictions (baseline); metric invariance (Model metric, MM), where equivalence restrictions are established between factor loads; scalar invariance (Model Scalar, MSca), that is, load and intercept equivalence restrictions; and strict invariance (Model Strict, MStr), taking into account the equivalence restrictions of factor loads, intercepts, and residuals. Invariance tests for gender, age (< 20 or > 20 years old, that is, the median of the sample) and type of university (public or private) were only planned for the best fitting model. We assessed the measurement invariance and its levels in accordance with the recommendations of Cheung and Rensvold (2002): ΔCFI ≤ .01 and ΔRMSEA ≤ .015 [30].

Thirdly, internal consistency was analyzed for the tested model with the most empirical support, based on the calculation of Cronbach’s α and McDonald’s ω, considering values greater than .80 acceptable for both cases [31]. This analysis includes internal consistency for the second-order factor (global score) and the four first-order factors.

Finally, the convergent validity was analyzed with Pearson’s correlation (*r*) between the PCQ-12 scores and that scale´s ratings related to academic engagement and satisfaction. The size of Pearson’s correlation (r) values can range from .10 to .29 (small), .30 to .49 (medium), and .50 to 1.0 (large) [32]. In particular, r = .50 to .69 represents a strong value, r = .70 to .89 represents a very strong value, and r ≥ .90 means that the relationship between the variables is perfect [33].

## Results

### Confirmatory factor analysis

With the aim of determining the PCQ- 12 factor structure, we compared the goodness-of-fit indexes of four distinct models of the Spanish version of the scale: one-factor structure (Model 1); first order four-factor structure (self-efficacy, hope, resilience and optimism) (Model 2); a four-factor model but of second order, similar to the first (Model 3); and a second-order 4-factor model but respecified taking into account the correlated errors between different items (Model 4).

The CMIN/DF, CFI, NFI, AIC, RMSEA, goodness-of-fit indexes of Models 1 to 3 were not as adequate as those presented by Model 4 (CMIN/DF = 7.99, CFI = .977, TLI = .970, NFI = .974, IFI = .980, AIC = 443.833, RMSEA = .064) (Table 1).

**Table 1:** Confirmatory factor analysis.

Models	CMIN/DF	CFI	TLI	NFI	IFI	AIC	RMSEA
M1	24.496	.912	.892	.909	.912	1370.758	.117
M2	8.424	.975	.966	.972	.975	464.344	.065
M3	9.537	.970	.961	.967	.970	532.870	.070
M4	7.997	.977	.970	.974	.980	443.833	.064

Note: M1(one factor); M2 (4 Factors first order); M3 (4 Factors Second order); M4 (4 Factors Second order respecified); minimum discrepancy per degrees of freedom (CMIN/DF); comparative fit index (CFI); Tucker Lewis index (TLI), the Bentler–Bonett normed fit index (NFI); incremental fit index (IFI), Akaike information criterion (AIC); root mean square error of approximation (RMSEA)

This empirical evidence suggests that the factorial structure that best explains the psychological capital scale is the second-order four-factor model respecified taking into account the correlations between items 1 and 2, 1 and 3, 2 and 3, 9 and 10, 9 and 11, 11 and 12 (see Fig 1).

The fit indexes of Model 4 (for both the total sample and separated by gender) are presented in Table 2, showing an adequate fit in each of them. On the one hand, the configural invariance (M1) presented good fit indicators (χ^2^ (104) = 461.892; CFI = .975 and RMSEA = .045 [.40 - .49]). Similarly, the metric invariance (M2) resulted in good fit indexes (χ^2^ (107) = 462.182; CFI = .976; RMSEA = .044 [.040 - .048]), being similar to the M1 values since they showed minimal differences (ΔCFI = .001 and ΔRMSEA = -.001). These results indicate that the factorial loads are invariant between the subsamples of men and women, and, therefore, the covariances can be compared. On the other hand, the scalar invariance (M3) demonstrated indexes equal to the previous model (χ^2^ (108) = 478.846; CFI = .974; RMSEA = .045 [.041 - .049]) with minimal differences (ΔCFI = -.002 and ΔRMSEA = 0.001), assuming the invariance between the different thresholds. The strict invariance (M4) reflects a good fit (χ^2^ (126) = 565.925; CFI = .970; RMSEA = .045 [.041 - .049]), showing no differences (ΔCFI = —.004 and ΔRMSEA = 0) and verifying the invariance of the residuals.

**Table 2:** Factorial invariance for the total sample and by gender, age and type of institution. Respecified second-order model.

Model	*χ* ^2^	df	C-M	Δχ^2^	Δ*df*	CFI	ΔCFI	SRMR	RMSEA	ΔRMSEA
Entire Group	383.833	48	-	-	-	.977	-	.053	.064	-
Men	159.684	48	-	-	-	.985	-	.048	.055	-
Women	274.012	48	-	-	-	.968	-	.062	0,070	-
M1	461.893	104	-	-	-	.975	-	.078	.045	-
M2	462.182	107	M2-M1	0.289	3	.976	.001	.079	.044	-.001
M3	478.846	108	M3 –M2	16.664	7	.974	-.002	.203	0,045	.001
M4	565.925	126	M4 –M3	87.079	0	.970	-.004	.202	.045	0
≤20years	252.756	48	-	-	-	.975	-	.056	.064	
≥21years	220.267	48	-	-	-	.973	-	.061	.072	-
M1	473.038	96	-	-	-	.974		.059	.048	-
M2	481.911	104	M2 –M1	8.873	8	.974	0	.069	.046	-.002
M3	483.867	107	M3 –M2	1.956	3	.974	0	.071	.045	-.001
M4	490.476	108	M4 –M3	6.609	1	.974	0	.144	.045	0
Public	193.632	48	-	-	-	.975	-	.056	.064	-
Private	232.587	48	-	-	-	.976	-	.059	0,067	-
M1	426.218	96	-	-	-	.975	-	.057	.046	-
M2	433.934	104	M2 –M1	7.716	8	.976	.001	.066	.044	-.002
M3	448.744	107	M3 –M2	14.81	3	.975	-.001	.079	0,044	0
M4	454.010	108	M4 –M3	5.266	1	.974	-.001	.118	.044	0

**Note:** chi squared (*X*^*2*^); comparison of factor invarance models (C-M); the Bentler comparative fit index (CFI); root mean square error of approximation (RMSEA).

Overall, the combined results demonstrated the factorial invariance of the Ecuadorian version of the PCQ-12 across gender (both male and female university students). Similar results were also obtained in relation to age and public and private institutions (see Table 2).

### Internal consistency and convergent validity

The internal consistency of Model 4 of the PCQ-12 scale showed satisfactory outcomes for both the total scale scores (α = .941, ω = .942) and its four factors: self-efficacy (α = .869, ω = .872), hope (α = .888, ω = .889), resilience (α = .774, ω = .785), and optimism (α = .840, ω = .840). These values can be seen in Table 3 as well as the mean score, standard deviation, and positive, high and statistically significant correlations between each of the factors that comprise psychological capital.

**Table 3:** Correlation between psychological capital, its dimensions, Cronbach’s alpha and McDonald’s ω.

	Psychological Capital	Self-efficacy	Hope	Resilience	Optimism
Psychological Capital		.873**	.934**	.871**	.868**
Self-efficacy			.763**	.646**	.667**
Hope				.732**	.770**
Resilience					.734**
Optimism					
Mean	53.68	13.84	18.14	12.51	9.17
Deviation	13.810	3.99	5.00	3.83	2.65
Cronbach’s Alfa	.941	.869	.888	.774	.840
McDonald´s ω	.942	.872	.889	.785	.840

Note: **p < 0.01.

Regarding convergent validity, we conducted correlation analyses between PCQ-12 scores and academic engagement and satisfaction. The main results (Table 4) showed positive and statistically significant (p < .01) relationships between the psychological capital scores and its factors with academic engagement and satisfaction, being the effect size high since they all range from .493 to .622.

**Table 4:** Convergent validity.

	Engagement	Satisfaction
Psychological capital	.622**	.609**
Self-efficacy	.530**	.540**
Hope	.597**	.577**
Resilience	.506**	.493**
Optimism	.583**	.553**

Note: *p < 0.01.

## Discussion

The objective of the study was to analyze the psychometric properties of the psychological capital scale adapted to Spanish in the academic context in university students from Ecuador, in order to address to the lack of validated and adapted instruments in Latin America. To our knowledge, no research had previously evaluated both the psychometric properties and factor structure in the Ecuadorian culture. In this study, it has been empirically verified, through construct validity, reliability (alpha and omega indexes) and convergent validity (Pearson’s correlations), that the PCQ-12 is a reliable and valid tool of academic psychological capital.

Regarding the construct validity, the results indicate that the factorial structure that best explains the psychological capital scale score interpretations is the second-order four-factor model respecified taking into account the correlations between the errors of items 1 and 2, 1 and 3, 2 and 3, 9 and 10, 9 and 11, 11 and 12. Likewise, this is consistent with the study carried out by Martínez et al. (2019) in two study samples, one Spanish and one Chilean, where they found a second-order model with 4 first-order factors and a better fit index by re-specifying the correlations between the errors of items 4 and 7. Therefore, there is a discrepancy between the correlations between the errors of the items. However, the results obtained in this research increase the external validity of those reported by Martinez et al. (2019).

On the other hand, the factorial structure obtained in this study differs from the one reported in students from Argentina and Brazil, where a model of four correlated factors is reported [18, 34]. Perhaps this inconsistency suggests that it is important to consider the cultural manifestations of PsyCap and the social factors that influence them.

Although, from a conceptual point of view, it seems that the four factors of PsyCap might overlap, the factorial structure found in this research, and in previous studies [18, 20, 34], suggests that self-efficacy, hope, optimism, and resilience are relevant and mutually exclusive dimensions that make up the psychological capital construct.

Regarding measurement invariance, results of our study showed that the second-order four-factor model respecified remained invariant across sex, age and public and private institutions. Researchers can proceed with examining the mean of those groups having confidence that, if any group differences are found, they are due to actual differences in psychological capital and not to an artefact of measurement error.

The internal consistency was adequate, with Alpha and Omega coefficients for the total scale (α = .941, ω = .942) and its four factors: self-efficacy (α = .869, ω = .872), hope (α = .888, ω = .889), resilience (α = .774, ω = .785), and optimism (α = .840, ω = .840), similar or even higher than those reported in previous research in the academic context [18, 20, 22, 27].

In terms of convergent validity, this study found positive and significant relationships with academic engagement and satisfaction. These results are consistent with other studies, in which psychological capital is positively and significantly correlated with academic engagement, school satisfaction [20, 35] and academic commitment [18].

Finally, the results derived from this research will allow Psychology professionals in countries where Spanish is the primary language to have an easy-to-use, 12-item instrument that measures psychological capital in an academic context, in a valid and reliable way.

### Limitations and practical implications

Several limitations of the study merit mention. First, our study should be considered with caution because the sample belongs only to undergraduate university students, but neither high school nor graduate students have been considered. Second, the age range has been very limited, and future studies should analyze the measure of invariances with a broader age range, as well as sociodemographic variables and those students who belong to other study modalities such as distance learning. Third, although the anonymity of the data was guaranteed and the information provided was used for research purposes only, the use of a self-report tool (such as the PCQ-12) may be subject to some inaccuracies in data collection due to possible memory biases, social desirability, and acquiescence responses [36]. For future research, we suggest the use of scales intended to measure and control potential biasing sources that may influence the results [37]. Finally, it is necessary to consider that this study corresponds to a non-probabilistic sample of university students.

Despite these limitations, the sample size and the values found empirically support our findings. In other words, the Spanish version of the PCQ-12 scale constitutes a reliable and valid instrument that will facilitate PsyCap research in university students in Ecuador or in countries in which Spanish is the primary language. The PsyCap seems to be multifarious and related to a wide variety of other positive psychological variables [38].

Also, the PCQ-12 scale is useful for evaluating intervention programs aimed at increasing psychological capital in academic contexts or other sectors where the focus is on student well-being [39]. The PsyCap is open to development and has already been proven to be alterable through interventions [38].

Additionally it can, represent an effective buffer against high levels of stress [38, 39, 40]. In this study, it was found that psychological capital is a second-order construct that involves the self-knowledge of being able to cope with academic demands (self-efficacy), the belief in positive outcomes (optimism), the ability to imagine alternative paths to overcome challenges (hope), and the ability to relate challenges and setbacks (resilience). Therefore, the PCQ-12 scale can be used to evaluate the PsyCap as a protective factor for students against stressful events related to academic activities and life transitions, such as leaving home, living with other people, loneliness, as well as social and financial problems [41].

## Conclusion

Psychological capital has been extensively researched in the workplace; however, there is little literature on it in the academic context. In this study, it has been found that the 12-item version in Spanish and adapted to the academic context has adequate psychometric properties, with a second order model composed of 4 factors proposed by the original authors (self-efficacy, optimism, hope and resilience). Therefore, it can be used for the evaluation of this construct within the academic framework.

## Supporting information

S1 Appendix Psychological capital questionnaire scale. (DOCX)

S1 Data (ZIP)
