# Supplementary material for: Psychometric properties and factorial structure of the Spanish version of the psychological capital scale in Ecuadorian university students
Source: PLoS One. 2023 May 25;18(5):e0285842. doi: 10.1371/journal.pone.0285842 (PMC10212118; doi:10.1371/journal.pone.0285842)
Supplement: S1 Appendix — (DOCX) [file pone.0285842.s001.docx]

**Appendix.**

**Psychological Capital Questionnaire: 12 Core Items**

BRIEF PSYCAP translated into Spanish and adapted to the academic context by Martinez et al. (20)

| 0 | 1 | 2 | 3 | 4 | 5 | 6 |
| --- | --- | --- | --- | --- | --- | --- |
| Completamente en desacuerdo | Parcialmente en desacuerdo | Algo en desacuerdo | Ni de acuerdo ni en desacuerdo | Algo de acuerdo | De acuerdo parcialmente | Completamente de acuerdo |

|  | Me siento seguro de mí mismo a la hora de expresar lo que opino de mis estudios |
| --- | --- |
|  | Me siento seguro de mí mismo a la hora de participar en conversaciones sobre las estrategias que deben guiar mis estudios |
|  | Me siento seguro al compartir información sobre mis estudios con otra gente |
|  | Si estuviese en apuros con mis estudios, se me ocurrirían muchas formas de salir adelante. |
|  | Actualmente creo que estoy teniendo bastante éxito en mis estudios. |
|  | Se me ocurren muchas formas de alcanzar mis actuales objetivos en los estudios. |
|  | En este momento, estoy logrando los objetivos que me he propuesto en mis estudios. |
|  | Si fuera necesario, podría “apañarme solo”, por así decirlo, en los estudios. |
|  | Normalmente, me tomo con calma los aspectos estresantes de mis estudios. |
|  | Puedo superar los momentos difíciles en mis estudios, porque ya me he enfrentado antes a las dificultades. |
|  | En lo que respecta a mis estudios, siempre miro el lado bueno de las cosas. |
|  | En lo que respecta a mis estudios, soy optimista en cuanto a lo que me deparará el futuro. |

Efficacy: Items 1-3

Hope: Items 4-7

Resilience: 8-10

Optimism: 11-12
